# Central and Peripheral Shoulder Fatigue Pre-screening Using the Sigma–Lognormal Model: A Proof of Concept

**DOI:** 10.3389/fnhum.2020.00171

**Published:** 2020-05-19

**Authors:** Anaïs Laurent, Réjean Plamondon, Mickael Begon

**Affiliations:** ^1^Laboratoire Scribens, Département de Génie Électrique, Programme de Génie Biomédical, Polytechnique Montréal, Montreal, QC, Canada; ^2^Laboratoire Scribens, Département de Génie Électrique, Polytechnique Montréal, Montreal, QC, Canada; ^3^Laboratoire de Simulation et de Modélisation du Mouvement, School of Kinesiology and Physical Activity Sciences, Faculty of Medicine, Université de Montréal, Montreal, QC, Canada; ^4^CHU Sainte-Justine, Montreal, QC, Canada

**Keywords:** Sigma–Lognormal model, Kinematic Theory of rapid human movement, central fatigue, peripheral fatigue, rotator cuff, handwriting, shoulder

## Abstract

**Background:**

Clinical tests for detecting central and peripheral shoulder fatigue are limited. The discrimination of these two types of fatigue is necessary to better adapt recovery intervention. The Kinematic Theory of Rapid Human Movements describes the neuromotor impulse response using lognormal functions and has many applications in pathology detection. The ideal motor control is modeled and a change in the neuromuscular system is reflected in parameters extracted according to this theory.

**Objective:**

The objective of this study was to assess whether a shoulder neuromuscular fatigue could be detected through parameters describing the theory, if there is the possibility to discriminate central from peripheral fatigue, and which handwriting test gives the most relevant information on fatigue.

**Methods:**

Twenty healthy participants performed two sessions of fast stroke handwriting on a tablet, before and after a shoulder fatigue. The fatigue was in internal rotation for one session and in external rotation during the other session. The drawings consisted of simple strokes, triangles, horizontal, and vertical oscillations. Parameters of these strokes were extracted according to the Sigma–Lognormal model of the Kinematic Theory. The evolution of each participant was analyzed through a *U-*Mann–Whitney test for individual comparisons. A Hotelling’s *T*^2^-test and a *U-*Mann–Whitney test were also performed on all participants to assess the group evolution after fatigue. Moreover, a correlation among parameters was calculated through Spearman coefficients to assess intrinsic parameters properties of each handwriting test.

**Results:**

Central and peripheral parameters were statistically different before and after fatigue with a possibility to discriminate them. Participants had various responses to fatigue. However, when considering the group, parameters related to the motor program execution showed significant increase in the handwriting tests after shoulder fatigue. The test of simple strokes permits to know more specifically where the fatigue comes from, whereas the oscillations tests were the most sensitive to fatigue.

**Conclusion:**

The results of this study suggest that the Sigma–Lognormal model of the Kinematic Theory is an innovative approach for fatigue detection with discrimination between the central and peripheral systems. Overall, there is a possibility to implement the setting for clinics and sports personalized follow-up.

## Introduction

One hundred million workers in the European population suffer from chronic musculoskeletal disorders and pain (Bevan, 2015). Direct and indirect costs for treating them are expensive, as they accounted, respectively, for up to $796.3 billion (which represents 5.2% of the national gross domestic product) and $130.7 billion in the US population per year between 2009 and 2011 (U.S. Bone and Joint Initiative, 2014). Shoulder is considered to be one of the most affected joints, as it represents the third cause of clinical consultation after the lumbar and cervical regions. Disorders at the rotator cuff in the shoulder region represents 50–85% of all shoulder musculoskeletal diseases in Québec (Roy et al., 2015). Overhead and arm elevation repetition movement is an important risk factor (Hagberg and Wegman, 1987; Svendsen et al., 2004; Ebaugh et al., 2006). In fact, while performing these movements, neuromuscular fatigue generates muscular and kinematic adaptations (Ebaugh et al., 2006; Gaudet et al., 2018), which can lead to musculoskeletal disorders (Beach et al., 1992). Sports requiring this kind of motion are then more affecting its players, like in volleyball, baseball, tennis, etc. (Wang and Cochrane, 2001; Mullaney et al., 2005; Wilk et al., 2009; Joshi et al., 2011). Detecting shoulder fatigue at an early stage could be a meaningful approach to avoid shoulder injuries.

Neuromuscular fatigue corresponds to “any exercise-induced loss of ability to produce force with a muscle or muscle group” (Taylor et al., 2006). It can be decomposed into two categories: central fatigue (Gandevia, 2001) and peripheral fatigue (Enoka and Stuart, 1992). Central fatigue implies the neural system: the voluntary activation and information conduction for movement execution are dysfunctional (Sesboüé and Guincestre, 2006; Boyas and Guével, 2011). Central fatigue can come from the supraspinal and spinal areas. Peripheral fatigue involves the muscles: in that case, the muscular excitation is impaired. It can cause for example a deterioration in the action potentials propagation or in the excitation–contraction coupling responsible for contraction (Sesboüé and Guincestre, 2006). A poor metabolite substrates supply can also be a consequence of peripheral fatigue, implying also an alteration of the excitation-contraction coupling (Boyas and Guével, 2011). The output force is reduced and the contractile mechanisms are dysfunctional (Bigland-Ritchie and Woods, 1984). In all cases, fatigue is different depending on the task (duration and weight lifted) and on the type of contraction (Chaffin et al., 2006a).

Several methods for detecting fatigue already exist. Numerous scales have been developed which are fatigue and task specific (Dittner et al., 2004). For example, the Visual Analog Scale is a reliable scale used to analyze a global fatigue and has already been used for muscular fatigue (Lee et al., 1991). However, this method is not so accurate for low intensities contractions (Leung et al., 2004). The Perceived Exertion Force is commonly used in fatigue studies, with the Borg’s scale (Borg, 1998) and in comparison to other scales, it seems to be one of the most accurate (Neely et al., 1992). However, results from this scale have to be analyzed carefully as it remains subjective (Chen et al., 2002). Objective approaches for fatigue detection also exist. One of the most frequently used is the electromyography (EMG) which was first employed by Piper (1912), according to Cifrek et al. (2009). Parameters such as the amplitude of the root mean square of the EMG signal increase with fatigue, as more motor units are recruited for the same amount of force produced (Merletti et al., 2004). The mean or median frequency of the power spectrum density decreases, as the velocity of action potentials is slowed down (Edwards and Lippold, 1956; Lindström et al., 1977; Al-Mulla et al., 2012). A complication with EMG is the quality of the signal to be assessed. It is essential to have good anatomical knowledge for electrodes placement, in order to avoid crosstalk problems as much as possible, which can lead to misinterpretations in the results analysis (Hermens et al., 2000; Merletti et al., 2001; Farina et al., 2004). EMG presents some difficulties for clinical evaluation as the electrode placement and signal treatment is time-consuming. There is also the possibility of using biomarkers as for example lactate concentrations (Tesch et al., 1978; Finsterer, 2012). Its intracellular concentration is supposed to diminish with the apparition of fatigue. Nevertheless, even if they are accurate methods, they remain invasive and hard to implement easily. Other non-invasive methods are employed for peripheral fatigue detection, such as sonomyography, near-infrared spectroscopy, mechanomyography, or acoustic myography (Mancini et al., 1994; Huang et al., 2007; Shi et al., 2007; Al-Mulla et al., 2011; Ibitoye et al., 2014). However, most of these techniques have to be synchronized with EMG to detect muscle fatigue and they cannot assess central fatigue. On the other hand, central fatigue can be evaluated either with percutaneous nerve stimulation –with an electrical nerve stimulation- or transcranial magnetic stimulation –with a nerve cells magnetic stimulation- (Gandevia, 2001; Taylor and Gandevia, 2001; Rozand et al., 2015) during maximal contractions. If the stimulation evokes an extra-force, it suggests that central fatigue is present (Merton, 1954). One more time, EMG can complement the methods to detect central and peripheral fatigue. Moreover, transcranial magnetic stimulation requires a magnetic coil to stimulate the motor cortex, which can interfere with EMG recordings (Valero-Cabré et al., 2011). In complement, percutaneous nerve stimulation is to our knowledge, so far not applicable to all muscles and requires an experimental set up lengthy and difficult to implement (Palmieri et al., 2004). This increases the risk of experimental misinterpretations, thus the difficulty to transpose it to a clinic. It is then necessary to find a method for detecting central and peripheral shoulder fatigue, which would be usable in clinics on a daily basis.

It has been shown that the Kinematic Theory of rapid human movements describes accurately the neuromotor control (Plamondon,1995a, b). This theory is based on the analysis of the velocity profiles of the end effector of a movement, like the finger, the wrist, the arm, the shoulder, the head, the trunk, the eye movements, etc. These movements can be modeled using lognormal functions, which depict the impulse response of the neuromuscular system of a participant (Plamondon et al., 2008). Thus, both central and peripheral information can theoretically be extracted from the movement reconstruction (Plamondon et al., 2003). In comparison, the Minimum-Jerk model (Hogan, 1984; Flash and Hogan, 1985) postulates that end effector trajectories are chosen by the central nervous system (CNS) such that the time integral of the squared magnitude of hand jerk is minimal, which is equivalent to maximizing the smoothness of the trajectory. Both approaches describe the same bell-shaped velocity using different analytical equations. Working with the Minimum-Jerk model does not give access to the command profile sent by the CNS. It is assumed that the alpha motoneuron signals, at the muscle level, correspond to a movement trajectory. This representation does not take into account the instant when the movement command is sent to the end-effector, neither the time required by the CNS to build and send the appropriate signals to the motor cortex neurons or the moment when the muscle starts to contract. The Minimum-Jerk does not give access to the central and peripheral information that we are investigating in this paper (Djioua and Plamondon, 2010). For these reasons, the Kinematic Theory was preferred to the Minimum-Jerk model for movement reconstruction. Moreover, the Kinematic Theory has been used in several pathologies for motor control studies, such as attention deficit hyperactivity disorder (Laniel et al., 2019), Parkinson’s disease (Lebel et al., 2017, 2018a,b; Nadeau et al., 2018), stroke risk factors (O’Reilly and Plamondon, 2011), concussion (Faci et al., 2020b), and it requires a non-invasive, low cost and plug-in-play experimental set-up made up of a digitizing tablet connected to a laptop. Its most recent implementation is ergonomic and very easy to use (Faci et al., 2018). Since during a shoulder fatigue the kinematic parameters and the fine motor control are modified (Qin et al., 2014), we hypothesized that the Kinematic Theory of rapid human movements may be relevant to monitor and assess shoulder fatigue analysis through graphomotricity. The objective of this work is to report a feasibility study aiming at the objective detection of muscular fatigue and the discrimination of central and peripheral fatigue, in an economical and non-invasive way, with the Kinematic Theory of rapid human movements.

## Materials and Methods

### Participants

Eleven males and nine females took part in the experiment. They were all healthy active adults (age: 23.2 ± 3.2 years, height: 173 ± 8.3 cm, mass: 71.7 ± 10.0 kg, 18 right-handed and 2 left-handed). All participants were free of any upper-limb musculoskeletal disorder and had no history of shoulder surgery or neurological disease in the past. The study was approved by the Research Ethics Committee of Polytechnique Montréal (CER-1819-23 v.3).

### Experimental Part

**FIGURE 1:**
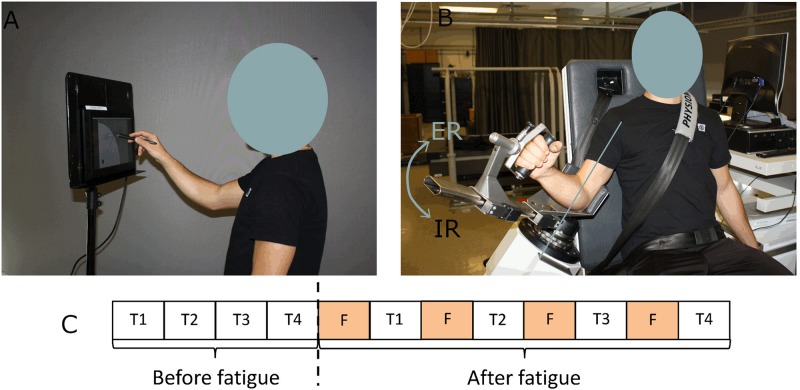
Experimental set-up. **(A)** Position of the participant while drawing strokes. **(B)** Setup of the participant on the dynamometer for the fatigue protocol. **(C)** Chronology of a session. T corresponds to a series of fast strokes (simple strokes, triangles, horizontal, and vertical oscillations) and F to a task of fatigue.

Participants completed two sessions in which they performed four series of fast strokes on a tablet before and after a task of shoulder fatigue. The two sessions were similarly performed, at the exception that the fatigue task targeted the shoulder external or internal rotators (sessions 1 and 2 randomly). There were at least 3 days of rest between the two sessions to avoid the participants to be still fatigued at the beginning of the second session. The process for each session was the following (Figure 1C): participants first had to execute the four series of fast strokes and then to alternate between a task of fatigue and a series of fast strokes. The series consisted of drawing simple strokes, triangles, horizontal oscillations and vertical oscillations in a random sequence.

The trajectory of fast strokes was recorded on a Wacom Cintiq 13HD tablet (Faci et al., 2018, 2020a). The tablet was positioned such that the participant’s fingertip touched the bottom of the tablet when the shoulder was 90° flexed. Participants had to position the stylus on the starting point of the tablet (Figure 1A). They started their movement as fast as possible at an audible stimulus (“bip” at 1 kHz for 500 ms) which was emitted after a random and unpredictable delay between 1 and 10 s. Depending on the task, a different guide-screen was displayed to help participants get the right movement [see further details regarding the protocol in O’Reilly et al. (2014)].

• (A) Simple strokes: participants were asked to draw 30 simple strokes from a starting point to a broad finish area. At the end of each stroke, participants had to maintain the stylus on the finish area for at least 1 s. A training period of 5–10 strokes was carried out before the recording.
• (B) Triangles: 30 triangles had to be drawn, passing through 3 points in the same clockwise or anticlockwise direction -chosen by the participant- and they had to wait with their stylus on the tablet for at least 1s at the end. A training period was also carried out before the recording.
• (C) Horizontal and (D) vertical oscillations: 10 s of oscillations at maximal speed between two parts spaced 50 mm apart were performed between two audible stimuli. After the second signal, stylus kinematics was still recorded until the participant completely stopped and maintained the stylus on the tablet for at least 1 s. Only one trial was registered in these cases, without any training period to avoid fatiguing participants with these two maximal speed tests.

The fatigue task consisted in repetitive submaximal dynamic contractions (concentric – continuous passive mode) at 90°/s (70° of amplitude) in internal or external rotation on an isokinetic dynamometer (CON-TREX^®^ MJ; Schnaittach, Germany). Participants were securely fastened using a belt so as not to move their back. Their arm was positioned at 30° of elevation (Figure 1B). A training period was allocated to familiarize the participant with the isokinetic effort and to warmup. To determine a target zone of 50 ± 7.5% of their maximum voluntary contraction, participants performed first a maximum voluntary isokinetic contraction in external rotation (or internal rotation during the other session). At each external rotation (or internal rotation) the participant was instructed to reach this target zone and to rest during the internal rotation (or external rotation). The Borg CR10 Scale (Borg, 1998) which varies from 0 (no effort at all) to 10 (the hardest exercise ever made) was asked every minute, to monitor perceived exertion. Stopping criteria of the fatigue trials were similar to those defined in Yang et al. (2018): (i) Borg number reached 9/10, (ii) three consecutive fails in reaching the target zone, (iii) after 30, 20, 15, and 10 min for the first, second, third, and fourth exercise of fatigue, respectively. The participants were not aware of these criteria. Verbal encouragements were provided as soon as the performance was outside the target zone. The number of Borg has been recorded for 19 participants in external rotation and 18 participants in internal rotation.

### Sigma–Lognormal Model

Data captured using the tablet were modeled according to the Kinematic Theory paradigm (Plamondon,1995a, b). This theory describes the velocity profile of an end effector as the synergetic impulse response of neuromuscular systems. Each of these systems is made of an infinite of subsystems, which are linked with a proportionality relationship between their cumulative time delays. From this postulate it is then predicted, according to the Central Limit Theorem (Plamondon et al., 2003) that the impulse response of a neuromuscular system tends toward a lognormal shape.

(1) $${\vec{v}}_{i}\,\left(t-t_{0}\right)={\vec{D}}_{i}\,\Lambda_{i}\,\left(t;t_{0\,i},\mu_{i},\sigma_{i}^{2}\right)$$

where *i* represents one lognormal, shifted with a time *t*_0_ with a command amplitude *D*; μ and σ representing timing properties of each lognormal such that:

(2) $$\Lambda_{i}\,\left(t;t_{0\,i},\mu_{i},\sigma_{i}^{2}\right)=\frac{1}{\sigma_{i}\,\sqrt{2\,\pi }\,\left(t-t_{0\,i}\right)}\,e\,x\,p\,\left\{-\frac{1}{2\,\sigma_{i}^{2}}\,{\left[\ln\left(t-t_{0\,i}\right)-\mu_{i}\right]}^{2}\right\}$$

In the case of a simple pointing task, the movement is seen as a synergy of two neuromuscular systems: an *agonist* and an *antagonist*. The *agonist* one is made up of muscles generating the desired action, whereas the *antagonist* system is made up of muscles working in the opposite direction of the desired movement. To that extent, *agonist* and *antagonist* lognormals can be distinguished based on the starting angle θ_si_ (see Equations 4 and 5). If the starting angle of the lognormal points toward the movement direction, the lognormal is *agonist*. If it points toward the opposite direction, the lognormal is *antagonist*. In that case, the resulting velocity can be expressed as the velocity of the *agonist* minus the *antagonist* lognormals. For more complex planar movements, the velocity can be described using a vector summation of lognormals. In that case, trajectories to reconstruct the movement are circle arcs which connect virtual targets defining an action plan. This means that the number of lognormals describing a movement corresponds to the number of virtual targets representing its trajectory (Plamondon and Djioua, 2006; O’Reilly and Plamondon, 2009).

(3) $$\vec{v}\,\left(t\right)=\sum_{i=1}^{N}{\vec{v}}_{i}\,\left(t;t_{0\,i},\mu_{i},\sigma_{i}^{2}\right)$$

(4) $$=\sum_{i=1}^{N}D_{i}\,\left[\begin{array}{c} c\,o\,s\,\left(\theta_{i}\,\left(t\right)\right)\\ s\,i\,n\,\left(\theta_{i}\,\left(t\right)\right) \end{array}\right]\,\Lambda_{i}\,\left(t;t_{0\,i},\mu_{i},\sigma_{i}^{2}\right);N\geq 2$$

(5) $$\theta_{i}\,\left(t\right)=\theta_{s\,i}+\frac{\left(\theta_{e\,i}-\theta_{s\,i}\right)}{2}\,\left[1+\text{erf}\,\left(\frac{\text{ln}\,\left(t-t_{0\,i}\right)-\mu_{i}}{\sigma_{i}\,\sqrt{2}}\right)\right]$$

These lognormal profiles have been observed and confirmed time and again in the last 15 years [see Plamondon (2020) for an extended survey (O’Reilly et al., 2013; Plamondon et al., 2013a)], which led to postulating and formalizing the guiding principle subtending the present research program: the Lognormality Principle (Plamondon et al., 2013b; Plamondon, 2020). According to this paradigm, the emergent lognormality of the neuromuscular impulse response of a given human motor system is a basic global feature reflecting the behavior of individuals who are in perfect control of their movements. The production of complex movements is accomplished by time superimposing and, summing up lognormal vectors, with the goal of minimizing their number in a given task, to produce efficient and fluent gestures and optimize the energy required to generate them. In this context, it is expected that neuromuscular fatigue will affect the lognormal parameters extracted from reconstructing a given set of gestures produced by a subject.

The main parameters describing a lognormal were extracted using an in-house software referred to as Script Studio (O’Reilly and Plamondon, 2009) and were splitted into four categories, which are resumed in the Supplementary Table S1 (Plamondon,1995b; Plamondon et al., 2003). Five parameters are regulated from the input level and they describe the central system command: (i) the time that takes the brain to perceive the stimulus and emit the command to the musculoskeletal system: *t*_0_ (s). It has to be differentiated to the stimulus onset, which is *T* = 0 s (O’Reilly et al., 2013) and the reaction time (RT) measured by the instant of movement onset. In other words, *t*_0_ refers to the moment when a population of neurons sends a motor command, it occurs after the audible stimulus is perceived and the motor command is prepared; (ii) Δ(*t*_0_) (s), which reflects the rhythmicity of an input command. It represents the time elapsed between two successive *t*_0_ and is used in the oscillations only; (iii) the amplitude of the lognormal command: *D* (mm), which corresponds to the distance covered by the resulting lognormal; (iv) the starting and (v) ending angles of the lognormal: θ*_s_* and θ*_e_* (rad). They describe the action plan made up of the lognormals.

Two parameters describe the timing properties of the neuromuscular system, in other terms the peripheral system of a participant: (vi) the long-time delay or the time taken to reach half of the distance movement on a logarithmic scale: μ [ln (s)]. It corresponds to the rapidity of a reaction to a command by a system; (vii) the log response time or the time taken from the neuromuscular system to respond to a command on a logarithmic scale: σ [ln(s)]. It is also linked to the movement duration and is a measure of the asymmetry of the lognormal.

The last two main parameters describe the global state of the neuromotor system: (viii) the number of lognormals required to reconstruct the velocity profile of the movement: *Nblog;* and (ix) the measure of the quality of the movement reconstruction, Signal-to-Noise Ratio: *SNR* (dB). They are completed with one derived parameter, (x) the *SNR/Nblog* (dB), that is used as a performance criterion and represents the motor control fluency of a gesture. The lognormality principle predicts that the ideal movement converges toward a lognormal profile. When the *SNR/Nblog* increases, the movement is closer to the ideal one, as postulated by the lognormal behavior (Plamondon et al., 2013b).

For our study, five derived parameters were also calculated for each type of strokes, representing the motor program execution (see equations in Supplementary Table S1). They give information about the velocity at which someone will react or execute a command, and the quality of its response: (xi) the *mode* (s), that is the time at which the maximum value of the lognormal impulse response is reached; (xii) the *median* (s), that is the time at which the half value of the integral under the lognormal curve (50% of the covered distance) is reached; (xiii) the *time delay* (s) which represents the rapidity of a neuromuscular system to respond to a command; (xiv) the *response time* (s) which is a measure of the spread of the impulse response; (xv) the *asymmetry* which characterizes the shape of the lognormal.

A last parameter, not from the theory, was also extracted: (xvi) the *reaction time* (RT) (s) that is the time needed to start the movement after a stimulus. In the present study, it was computed as the time required to reach 10% of the maximal velocity during the test. From this parameter, we calculated (xvii) *RT-t_0_* (s) which is the duration of the command propagation (Woch and Plamondon, 2001).

### Data Formatting

**FIGURE 2:**
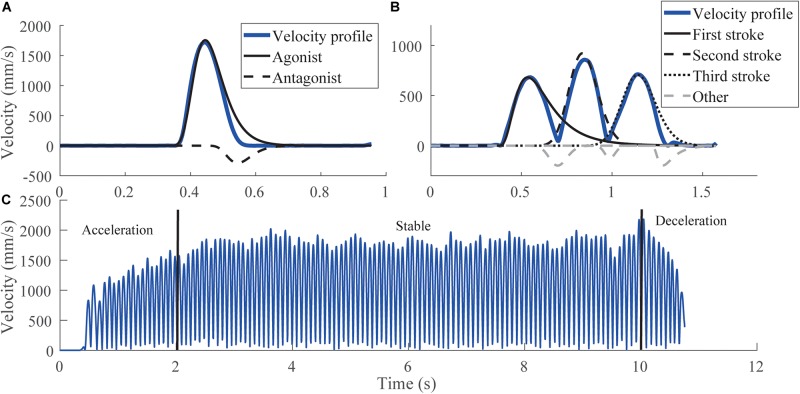
Different categories of Lognormals for each test. **(A)** Original velocity profile (blue) of a simple stroke, with its decomposition into *agonist* and *antagonist* components. **(B)** Original velocity profile (blue) of a triangle with an extraction of the Lognormal corresponding to the first, second, or third stroke. Dashed gray lines correspond to other Lognormals used for reconstruction but not analyzed. **(C)** Original velocity profile (blue) of the oscillations, with the three-phase separation. The reconstruction of the velocity profile for oscillations is similar to the one of triangles, except that there are only *agonist* Lognormals, as the movement is fluent and stops only after the 10 s.

The lognormals extracted from each test were split into components as follows. For the simple strokes, two lognormals that defined the largest *agonist* and largest *antagonist* components were analyzed (Figure 2A). Strokes composed by only one lognormal were classified as *agonists* (Laurent et al., 2019). For the triangles, strokes were decomposed into the three largest lognormals explaining stroke 1, stroke 2, and stroke 3 (Figure 2B). It was manually checked that triangles were properly reconstructed. Those whose lognormals did not describe their correct trajectory where rejected. It is noticed when the starting angle of the reconstruction did not point toward the stroke direction. For oscillations, strokes were split into three phases (Figure 2C): acceleration (0–2 s), stable (2–10 s), and deceleration (10 s and more) phases. Lognormals whose amplitude was lower than 50 mm were considered as artifacts and rejected. The remaining lognormals were then classified according to the gesture performed. For the horizontal oscillations (vertical oscillations in the other case), if the cosine (sine in the other case) of the starting angle was positive, the lognormals were considered as an external rotation movement, otherwise they were considered as an internal rotation movement. For each type of strokes, and each participant, lognormals having at least one parameter outside the mean ± 3SD were rejected. The proportion of lognormals retained by tests is reported in Section “Results.”

### Statistical Analyses

To assess the evolution of each participant after fatigue, individual comparisons using a paired *U*-Mann–Whitney test (non-parametric paired *t-*test) were completed. This test was performed using all the Lognormals of the 30 strokes and depending on the type of fatigue (ER or IR). For the simple strokes and the triangles, 16 parameters were compared, the statistical significance level was thus set at *p* < 0.00031 (i.e., 0.05/16) after Bonferroni correction. For the oscillations, *SNR*, *SNR/Nblog*, and *Nblog* were not analyzed as there was only one value with the oscillations, the significance level was set at *p* < 0.0042 (i.e., 0.05/12).

For group comparisons (*n* = 20) t_0_, *D*, μ, σ, *|cos*(θ*_s_*)*|*, *|cos*(θ*_e_*)*|*, *mode*, *median*, *time delay*, *response time*, *Nblog*, *SNR*, and *SNR/Nblog* were chosen for the simple strokes and triangles. For the oscillations, Δ(*t*_0_), *D*, μ, σ, *mode*, *median*, *time delay*, *response time*, *Nblog*, *SNR*, and *SNR(dB)/Nblog* were selected. Parameters of the oscillations were extracted from the stable phase. Only the *SNR* and *SNR/Nblog* were calculated from the whole signal. Due to signals recording problems, the data of four participants were rejected for the analysis of the *Nblog*, *SNR*, and *SNR/Nblog* for the vertical oscillations during an internal rotation fatigue and of one participant, for the horizontal oscillations during an internal rotation fatigue.

A non-parametric paired Hotelling’s *T*^2^-test on each series of fast strokes was first performed including all the parameters. This multivariate test assessed whether there are statistical differences between the two conditions (without and with fatigue) considering all the parameters. When the test was statistically significant (*p* < 0.05), the non-parametric paired *U*-Mann–Whitney test was performed on each parameter separately. The statistical significance was set at *p* < 0.00385 (0.05/13) for the simple strokes and the triangles and at *p* < 0.0042 (0.05/12) for the oscillations. Comparisons were performed on all lognormals, considering separately *agonist* and *antagonist* components for the simple strokes, except for the *Nblog*, *SNR*, and *SNR/Nblog*, as the whole signal was considered. No such distinctions between lognormals were made for the triangles and the oscillations since no supplementary information could be assessed. The Cohen’s d effect size was also calculated to estimate the importance of the parameters evolution after fatigue. As referred in Sawilowsky (2009) the description for magnitude is the following: *d*(0.01) = very small, *d*(0.20) = small, *d*(0.50) = medium, *d*(0.80) = large, *d*(1.20) = very large, and *d*(2.0) = huge.

Correlation matrices were finally calculated to assess the relationships between parameters. The correlation between the *reaction time* and *t*_0_ was assessed to determine the importance of using *t*_0_ for central system analyses and the correlation of *t*_0_ with μ to evaluate the independence of parameters related to the central and peripheral systems. To do so, Spearman coefficients were evaluated on the mean of each parameter by test. Statistical significance was set at *p* < 0.05.

## Results

**FIGURE 3:**
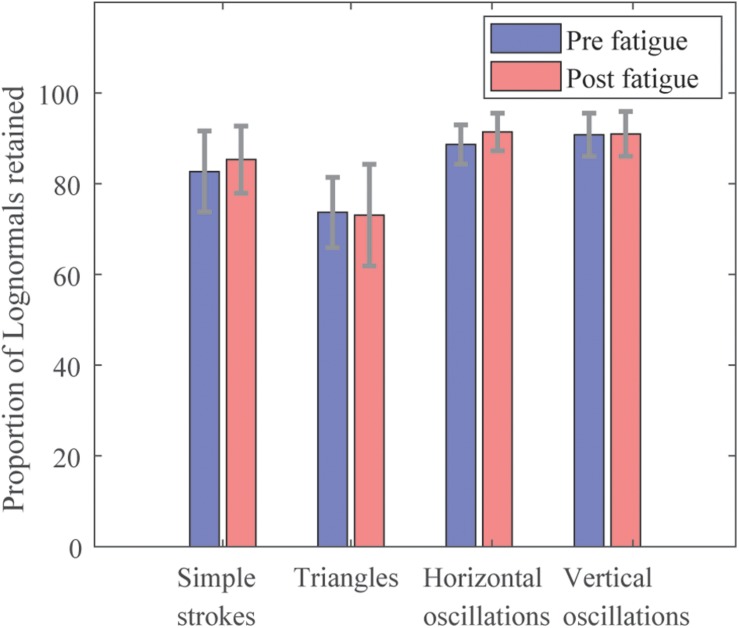
Proportion of Lognormals retained by test.

The filtering of data led to retain lognormals with properties verifying the conditions mentioned in Section “Data Formatting.” The proportion of the lognormals retained out of the entire set of strokes drawn by test, is illustrated in Figure 3. The proportions are similar pre- and post-fatigue. It is observed that horizontal and vertical oscillations have the highest amount of lognormals retained (between 88.7 ± 0.04% and 91.4 ± 0.04%), whereas triangles count the lowest numbers of them (73.7 ± 0.08% pre-fatigue versus 73.1 ± 0.11% post-fatigue). Simple strokes have around 84% of lognormals retained.

### Effects of Fatigue by Participant

#### Torques and Borg Number

**FIGURE 4:**
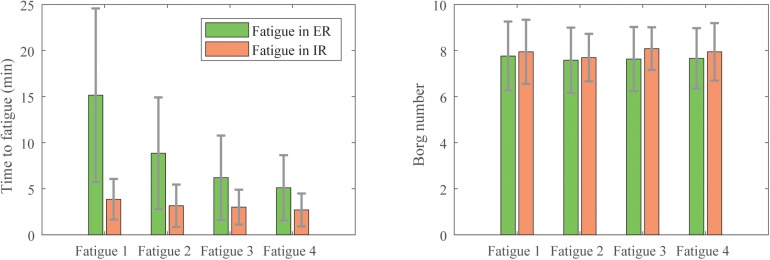
Means and standard deviations of the duration of fatigue **(Left)** and the perceived exertion on the Borg scale **(Right)** of the participants during each of the four fatigue sessions.

As depicted in Figure 4, the participants experienced internal rotation (IR) or external rotation (ER) fatigue differently. The ER fatigue trials lasted longer than the IR ones (15 ± 9.4 min versus 3.9 ± 1.6 min). We observed also that the time necessary to fatigue after each series of strokes decreased for the ER fatigue (respectively, 15, 8.9, 6.2, and 5.1 min) whereas it stayed quite stable for the IR fatigue (3.9, 3.2, 3, and 2.7 min). However, for both fatigues, the number on the Borg Scale was similar (7.7 ± 1.4 for the ER fatigue and 7.9 ± 1.1 for the IR fatigue). Despite the shorter time to fatigue, participants perceived intense effort in IR.

#### Kinematic Parameters

**FIGURE 5:**
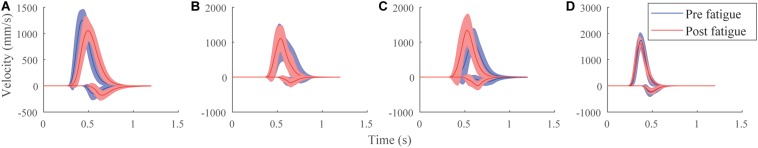
Velocity profiles of four participants with the mean ± SD of the simple strokes, before (blue) and after (red) fatigue for the *agonist* (positive) and *antagonist* (negative) components. Panel **(A)** had a significant increase after fatigue in μ, *mode*, *median*, *time delay*, *RT* for the *agonist* parameters; and *t*_0_, σ, *response time*, *asymmetry*, and *RT* for the *antagonist* parameters. Panel **(B)** had no statistical changes. Panel **(C)** had a significant increase in *D* for the *agonist* parameters. It had a significant decrease in *|cos*(θ*_s_*)*|*, μ, *mode*, *median*, *time delay*, *Nblog* in the *agonist* parameters and *SNR* and *TR* in both components. Panel **(D)** had a significant increase after fatigue of *t*_0_, σ and *|t_0_-RT|* and a significant decrease in μ and *RT* in the *agonist* parameters.

The proportion of participants affected in their parameters by fatigue is presented in Table 1. Both central and peripheral systems were affected by fatigue in each stroke of each test, as preliminary reported in Laurent et al. (2019), for both ER or IR fatigue. Parameters affected by fatigue were not necessarily the same among all participants and neither was their evolution. This inter-subject variability is illustrated in Figure 5, where four velocity profiles are drawn pre- and post-fatigue, characterizing different participant’s behavior. For example, after fatigue, the velocity profile was either displaced to the right (Figure 5A), to the left (Figure 5C), or not evolving (Figures 5B,D).

**TABLE 1:** Percentage of participants (*N* = 20) with significant differences for each component of their tests.

Test	Stroke	Central system	Peripheral system	Both systems	Motor program execution	Global state of the neuromotor system	RT	|*t*_0_-RT| or |Δ(*t*_0_)-RT|
**Fatigue in external rotation (ER)**								
Simple strokes	*Agonist*	70	40	30	40	50	45	5
	*Antagonist*	60	15	10	15	45		
	Total	90	40	35	45	55		
Triangles	Stroke 1	45	25	10	55	10	20	15
	Stroke 2	45	20	5	65			
	Stroke 3	25	5	5	55			
	Total	75	30	20	95			
Horizontal oscillations	External rotation	95	80	75	80	x	x	70
	Internal rotation	100	70	70	85			
	Total	100	75	85	95			
Vertical oscillations	External rotation	90	55	55	65	x	x	95
	Internal rotation	90	35	35	75			
	Total	95	65	60	80			
**Fatigue in internal rotation (IR)**								
Simple strokes	*Agonist*	95	20	20	25	50	30	10
	*Antagonist*	30	20	15	25	45		
	Total	95	35	30	45	55		
Triangles	Stroke 1	30	15	5	50	0	10	0
	Stroke 2	20	25	10	65			
	Stroke 3	45	20	20	70			
	Total	75	40	25	85			
Horizontal oscillations	External rotation	95	80	80	90	x	x	85
	Internal rotation	100	75	75	90			
	Total	100	85	85	95			
Vertical oscillations	External rotation	90	75	65	80	x	x	85
	Internal rotation	100	65	65	90			
	Total	100	85	85	90			

*We could not perform t-test for the parameters reflecting the global state of the neuromotor system and the reaction time (RT) for the oscillations tests as we have only one value per subject. For the oscillations |Δ(t_0_)-RT| was reported instead of |t_0_-RT|.*

For the simple strokes, 90% of the participants had almost one parameter describing their central system significantly different post ER fatigue, either in the *agonist* or *antagonist* component (Table 1). It was changed in 95% of the population after an IR fatigue. For the parameters reflecting the peripheral system, more differences were noticed in the *agonist* parameters (40%) than in the *antagonist* parameters (15%) after an ER fatigue. After an IR fatigue, no such distinction between *agonist* and *antagonist* parameters was found for the peripheral system (20% of statistical changes in both cases). The conduction time was affected in only 5% of the population after an ER fatigue and 10% after an IR fatigue.

For the triangles, the global state of the neuromotor system was impacted in 10% of the population after an ER fatigue and in no participant at all after IR fatigue. The motor program execution showed numerous differences pre- and post-fatigue for the triangles, as in average more than 85% of the population presented statistical differences (85% after an IR fatigue and 95% after an ER fatigue). The conduction time *|t_0_-RT|* was affected in 15% of the population after an ER fatigue, and in no participants after an IR fatigue.

The oscillations were the tests in which the most significant differences were observed pre- and post-fatigue. All participants had statistical changes in the parameters related to the central system and the motor program execution, both after ER or IR fatigue. Moreover, after an ER fatigue, peripheral parameters changed in 75% of the population in horizontal oscillations *versus* 65% in the vertical oscillations. After an IR fatigue, they changed in 85% both in horizontal and vertical oscillations.

### Group Effect of Fatigue

#### Parameters Evolution

Hotelling’s *T*^2^-tests were all statistically significant (*p* < 0.05), except for the triangles after an IR fatigue. As a matter of fact, they validated in those cases the use of the *U*-Mann–Whitney test to assess each parameter evolution. For the *agonist* component of simple strokes, σ and the *time delay* were significantly higher after fatigue (*p* = 0.0001), with a medium and large effect size (*d* = 0.66 and 0.93) (Table 2). In the *antagonist* components *t*_0_ and the *response time* were significantly higher after fatigue (*p* = 0.0001), with a medium effect size (*d* = 0.51 and 0.56, respectively). The *SNR/Nblog* significantly decreased after fatigue (*p* = 0.0002). After an IR fatigue, *t*_0_ was significantly higher after fatigue only for the *agonist* components (*agonist*, *p* = 0.0002; *antagonist*, *p* = 0.7). The *mode*, *median* and *time delay* increased for both components (*p* ≤ 0.0002), with medium effect size, ranging from 0.53 to 0.79. In the triangles, *D*, the *mode*, the *median* and the *time delay* were significantly higher after an ER fatigue (*p* = 0.0001) (Table 3). For the horizontal oscillations (Table 4), Δ(*t*_0_) was significantly higher after an ER fatigue with a large effect size (*d* = 0.80). Regarding the peripheral system, μ increased after both an ER and IR fatigue (*p* = 0.0001), with a large effect size (*d* = 0.82 and 1.21, respectively). The *mode*, *median*, *time delay* and *response time* were significantly higher (*p* = 0.0001) with a large effect size after ER and IR fatigues (*d* = 0.80–1.18). The *SNR/Nblog* was significantly higher after an IR fatigue, with a medium effect size (*d* = 0.61). In the vertical oscillations, *D* was significantly higher (*p* = 0.0001), after an ER fatigue. After an IR fatigue the *mode*, *median*, *time delay*, and *response time* were significantly higher (*p* = 0.0001), with a medium effect size (*d* = 0.58-0.61).

**TABLE 2:** Parameters evolution in the simple strokes after a shoulder fatigue in external or internal rotation.

		*Agonist*				*Antagonist*			
		Pre-fatigue	Post-fatigue	*P*-value	Effect size	Pre-fatigue	Post-fatigue	*P*-value	Effect size

External rotation fatigue (*N* = 20)									
**Central system**									
	***t*_0_**	0.23 ± 0.08	0.25 ± 0.07	0.0246	0.32	0.40 ± 0.12	0.44 ± 0.12	0.0002*	0.51^a^
	***D***	214 ± 19.8	217 ± 21.6	0.005	0.20	30.6 ± 6.94	33.0 ± 8.33	0.0001*	0.33
	**|cos(θ_s_)|**	0.81 ± 0.08	0.81 ± 0.10	0.0001*	0.15	0.95 ± 0.03	0.94 ± 0.03	0.0001*	0.31
	**|cos(θ_e_)|**	0.96 ± 0.03	0.96 ± 0.03	0.0342	0.33	0.91 ± 0.11	0.94 ± 0.05	0.0001*	0.36
**Peripheral system**									
	**μ**	−1.42 ± 0.18	−1.41 ± 0.15	0.4648	0.02	−1.78 ± 0.18	−1.82 ± 0.20	0.3042	0.23
	**σ**	0.27 ± 0.06	0.30 ± 0.06	0.0001*	0.66^a^	0.36 ± 0.11	0.39 ± 0.12	0.0004*	0.47
**Motor program execution**									
	**Mode**	0.47 ± 0.08	0.48 ± 0.08	0.0002*	0.28	0.59 ± 0.10	0.62 ± 0.10	0.0001*	0.41
	**Median**	0.49 ± 0.08	0.51 ± 0.08	0.0001*	0.33	0.61 ± 0.11	0.64 ± 0.11	0.0001*	0.45
	**Time delay**	0.50 ± 0.09	0.51 ± 0.09	0.0001*	0.36	0.62 ± 0.11	0.65 ± 0.11	0.0001*	0.47
	**Response time**	0.07 ± 0.02	0.08 ± 0.02	0.0001*	0.93^b^	0.06 ± 0.03	0.07 ± 0.03	0.0001*	0.56^a^
**Global state of the neuromotor system – whole stroke**									
	**Nblog**	2.18 ± 0.26	2.29 ± 0.30	0.0002*	0.48				
	**SNR**	30.3 ± 1.14	29.7 ± 1.29	0.0001*	0.44				
	**SNR/Nblog**	14.7 ± 1.53	13.8 ± 1.90	0.0002*	0.52^a^				

**Internal rotation fatigue (*N* = 20)**									

**Central system**									
	***t*_0_**	0.21 ± 0.08	0.24 ± 0.08	0.0002*	0.52^a^	0.40 ± 0.11	0.41 ± 0.11	0.7018	0.20
	***D***	208 ± 25.0	208 ± 23.7	0.713	0.00	29.0 ± 6.66	30.4 ± 7.53	0.2932	0.23
	**|cos(θ_s_)|**	0.81 ± 0.09	0.79 ± 0.09	0.0001*	0.65^a^	0.93 ± 0.06	0.94 ± 0.04	0.7014	0.25
	**|cos(θ_e_)|**	0.95 ± 0.03	0.96 ± 0.02	0.0001*	0.44	0.91 ± 0.09	0.93 ± 0.03	0.0004*	0.47
**Peripheral system**									
	**μ**	−1.43 ± 0.18	−1.46 ± 0.18	0.0154	0.27	−1.86 ± 0.22	−1.80 ± 0.16	0.0454	0.24
	**σ**	0.27 ± 0.05	0.29 ± 0.05	0.0001*	0.40	0.36 ± 0.08	0.36 ± 0.10	0.2972	0.06
**Motor program execution**									
	**Mode**	0.45 ± 0.08	0.47 ± 0.09	0.0002*	0.53^a^	0.57 ± 0.10	0.59 ± 0.10	0.0002*	0.79^a^
	**Median**	0.47 ± 0.09	0.49 ± 0.09	0.0001*	0.58^a^	0.59 ± 0.10	0.61 ± 0.11	0.0001*	0.73^a^
	**Time delay**	0.48 ± 0.09	0.50 ± 0.10	0.0001*	0.58^a^	0.60 ± 0.11	0.63 ± 0.12	0.0001*	0.67^a^
	**Response time**	0.07 ± 0.02	0.07 ± 0.02	0.0001*	0.40	0.06 ± 0.02	0.06 ± 0.03	0.0492	0.17
**Global state of the neuromotor system – whole stroke**									
	**Nblog**	2.20 ± 0.24	2.20 ± 0.25	0.9086	0.02				
	**SNR**	30.1 ± 1.12	30.0 ± 1.37	0.7352	0.09				
	**SNR/Nblog**	14.5 ± 1.52	14.5 ± 1.77	0.9802	0.00				

The simple strokes are separated into their agonist or antagonist components. Values are expressed as mean ± standard deviation. *Represents statistical significance (p < 0.05/13 = 0.0038). ^*a,b*^Represent medium and large effect sizes, respectively.

**TABLE 3:** Parameters evolution in the triangles, after a shoulder fatigue in external rotation.

	Triangles – whole stroke (*N* = 20)			
	Pre-fatigue	Post-fatigue	*P*-value	Effect size
**Central system**				
***t*_0_**	0.23 ± 0.08	0.23 ± 0.06	0.488	0.12
***D***	154.9 ± 9.29	158.0 ± 10.2	0.0001*	0.53^a^
**Peripheral system**				
**μ**	−0.80 ± 0.21	−0.77 ± 0.18	0.0028*	0.31
**σ**	0.19 ± 0.03	0.19 ± 0.03	0.0226	0.25
**Motor program execution**				
**Mode**	0.74 ± 0.08	0.75 ± 0.08	0.0001*	0.40
**Median**	0.75 ± 0.09	0.77 ± 0.08	0.0001*	0.38
**Time delay**	0.76 ± 0.09	0.77 ± 0.08	0.0001*	0.36
**Response time**	0.08 ± 0.01	0.08 ± 0.01	0.6214	0.01
**Global state of the neuromotor system**				
**Nblog**	5.18 ± 0.61	5.21 ± 0.38	0.6678	0.06
**SNR**	27.0 ± 0.30	26.8 ± 0.47	0.0034*	0.46
**SNR/Nblog**	5.37 ± 0.69	5.37 ± 0.45	0.7124	0.01

Parameters come from combined strokes. Values are expressed as mean ± standard deviation. *Represents statistical significance (p < 0.05/11 = 0.0045). ^*a*^Represents medium effect size.

**TABLE 4:** Parameters evolution in the horizontal and vertical oscillations, after a shoulder fatigue in external or internal rotation.

Horizontal oscillations (*N* = 20)									
		External rotation fatigue				Internal rotation fatigue			
		Pre-fatigue	Post-fatigue	*P*-value	Effect size	Pre-fatigue	Post-fatigue	*P*-value	Effect size
**Central system**									
	**Δ(*t*_0_)**	0.08 ± 0.01	0.09 ± 0.01	0.0012*	0.80^b^	0.09 ± 0.01	0.09 ± 0.01	0.0150	0.96
	***D***	124.8 ± 23.7	126.2 ± 26.2	0.0008*	0.10	124.2 ± 24.5	125.2 ± 28.8	0.2290	0.04
**Peripheral system**									
	**μ**	−0.81 ± 0.07	−0.75 ± 0.10	0.0001*	0.82^b^	−0.81 ± 0.11	−0.75 ± 0.10	0.0001*	1.21^c^
	**σ**	0.06 ± 0.00	0.06 ± 0.00	0.8870	0.09	0.06 ± 0.00	0.06 ± 0.00	0.0568	0.32
**Motor program execution**									
	**Mode**	0.53 ± 0.04	0.56 ± 0.06	0.0001*	0.80^b^	0.53 ± 0.06	0.57 ± 0.06	0.0001*	1.18^b^
	**Median**	0.53 ± 0.04	0.57 ± 0.06	0.0001*	0.80^b^	0.53 ± 0.06	0.57 ± 0.06	0.0001*	1.18^b^
	**Time delay**	0.53 ± 0.04	0.57 ± 0.06	0.0001*	0.80^b^	0.53 ± 0.06	0.57 ± 0.06	0.0001*	1.18^b^
	**Response time**	0.03 ± 0.02	0.03 ± 0.00	0.0001*	0.81^b^	0.03 ± 0.00	0.03 ± 0.00	0.0001*	1.17^b^
**Global state of the neuromotor system**									
	**Nblog**	94.7 ± 6.78	89.1 ± 8.79	0.0002*	0.76^a^	94.7 ± 10.4	89.4 ± 9.21	0.0002*	0.66^a^
	**SNR**	28.4 ± 1.40	27.6 ± 1.26	0.0868	0.38	28.4 ± 1.12	28.2 ± 0.99	0.3824	0.10
	**SNR/Nblog**	0.21 ± 0.02	0.23 ± 0.03	0.0068	0.56^a^	0.21 ± 0.03	0.23 ± 0.02	0.0002*	0.61^a^

**Vertical oscillations (*N* = 20)**									
**Central system**									
	**Δ(*t*_0_)**	0.09 ± 0.01	0.09 ± 0.01	0.7646	0.09	0.09 ± 0.02	0.10 ± 0.01	0.0256	0.56
	***D***	118.4 ± 19.2	123.9 ± 19.4	0.0001*	0.57^a^	119.4 ± 26.0	119.8 ± 21.6	0.4200	0.03
**Peripheral system**									
	**μ**	−0.73 ± 0.11	−0.73 ± 0.14	0.9816	0.05	−0.74 ± 0.17	−0.69 ± 0.16	0.0001*	0.64^a^
	**σ**	0.06 ± 0.00	0.06 ± 0.00	0.6778	0.04	0.06 ± 0.00	0.06 ± 0.00	0.1486	0.23
**Motor program execution**									
	**Mode**	0.57 ± 0.07	0.58 ± 0.09	0.4820	0.11	0.58 ± 0.10	0.61 ± 0.09	0.0001*	0.61^a^
	**Median**	0.58 ± 0.07	0.58 ± 0.09	0.4854	0.11	0.59 ± 0.10	0.61 ± 0.09	0.0001*	0.61^a^
	**Time delay**	0.58 ± 0.07	0.58 ± 0.09	0.4906	0.11	0.59 ± 0.10	0.61 ± 0.09	0.0001*	0.61^a^
	**Response time**	0.03 ± 0.00	0.028 ± 0.004	0.1838	0.11	0.03 ± 0.01	0.03 ± 0.004	0.0001*	0.58^a^
**Global state of the neuromotor system**									
	**Nblog**	88.0 ± 9.51	88.1 ± 12.1	0.9334	0.03	88.4 ± 14.9	84.8 ± 12.1	0.0314	0.40
	**SNR**	27.6 ± 1.81	27.9 ± 1.46	0.5268	0.15	28.0 ± 1.64	27.4 ± 1.39	0.3432	0.27
	**SNR/Nblog**	0.22 ± 0.02	0.23 ± 0.04	0.3886	0.20	0.23 ± 0.04	0.24 ± 0.04	0.6482	0.18

Parameters come from combined strokes. Values are expressed as mean ± standard deviation. For the global state of the neuromotor system, N = 19 after an ER fatigue and N = 16 after an IR fatigue. *Represents statistical significance (p < 0.05/11 = 0.0045). ^*a,b,c*^Represent medium, large and very large effect sizes, respectively.

#### Correlation Between Parameters

The reaction time (*RT*) was correlated with *t*_0_ of the *agonist* and *antagonist* component of the simple strokes (Table 5). The correlation with the triangles existed only with the *t*_0_ of the first stroke. These correlations were present pre- and post-fatigue (ρ from 0.78 to 0.89 for the triangles and from 0.72 to 0.93 for the simple strokes). Parameters were more correlated for the *agonist* component than the *antagonist*. For example, before ER fatigue, the correlation was set at ρ = 0.88 for the *agonist* component and at ρ = 0.76 for the *antagonist* component. Regarding *t*_0_ and μ, there is no evidence of high correlation for the simple strokes (ρ between −0.51 and −0.08 depending on the test) but it appears for the triangles (ρ from −0.86 to −0.47).

**TABLE 5:** Spearman correlation coefficients between parameters, with rho-values of correlation pre- and post-fatigue.

Parameters	Test	Type of fatigue	Type of stroke	Rho pre-fatigue	Rho post-fatigue
***t*_0_ and RT**	Simple strokes	ER	*Agonist*	0.88*	0.93*
			*Antagonist*	0.76*	0.72*
		IR	*Agonist*	0.91*	0.92*
			*Antagonist*	0.86*	0.74*
	Triangles	ER	Stroke 1	0.89*	0.78*
			Stroke 2	0.07	0.20
			Stroke 3	–0.02	0.15
		IR	Stroke 1	0.78*	0.81*
			Stroke 2	0.23	–0.10
			Stroke 3	0.10	0.24
***t*_0_ and μ**	Simple strokes	ER	*Agonist*	–0.37	–0.19
			*Antagonist*	–0.42	−0.51*
		IR	*Agonist*	–0.08	–0.21
			*Antagonist*	–0.14	–0.18
	Triangles	ER	Stroke 1	−0.74*	−0.61*
			Stroke 2	−0.86*	−0.83*
			Stroke 3	−0.74*	−0.85*
		IR	Stroke 1	−0.58*	−0.47*
			Stroke 2	−0.83*	−0.82*
			Stroke 3	−0.76*	−0.52*

*Represents significant correlations (p < 0.05).

## Discussion

This study aimed to settle an innovative, economical and non-invasive method to detect shoulder muscular fatigue and discriminate between central and peripheral fatigue.

### Distinction of the Type of Fatigue

In the Kinematic Theory of rapid human movements, the distinction between central and peripheral fatigue is possible through the intrinsic properties of the parameters extracted from each stroke (O’Reilly and Plamondon, 2013). In addition to these parameters, we proposed a series of derived parameters, which translate a more global approach of motor control analysis. For the oscillations, using Δ(*t*_0_) instead of *t*_0_ seems relevant for the central system analysis. As *t*_0_ represents the timing emission for a command (Plamondon,1995b), Δ(*t*_0_) describes the frequency at which the emission command is sent. A statistical change means that the brain rapidity for generating command signals is impaired due to fatigue. On the other hand, the peripheral system is reflected through μ and σ, which are the temporal properties of the neuromuscular system. A significant difference in one of these parameters theoretically means that the peripheral system of the participant was impaired by fatigue. As exposed in Table 1, all the tests performed could reflect those changes. In practice, the correlation between the *reaction time* and *t*_0_ (Table 5) consolidated our position of using *t*_0_ for the central nervous system analysis. In fact, the *reaction time* is a commonly used parameter for cognitive studies (Tanaka et al., 2009; Sant’Ana et al., 2017). This correlation was higher for the first stroke of the simple strokes and the triangles. As the *antagonist* component appears after the *agonist* one, there is a delay, so a lower correlation. The same remark can be made for the triangles: strokes 2 and 3 appear later implying an absence of correlation between *RT* and their *t*_0_. In addition, the calculation of the conduction time *|t_0_-RT|* enables to locate more precisely the origin of the central fatigue. In our study, it changed for a small population (≤15%), whether simple strokes or triangles. This means that the time taken from the brain to propagate the information to the end effector does not change for most of the participants. Moreover, for clinical purposes, it would be of interest to differentiate the fatigued muscle, whether the *infraspinatus* (ER fatigue) or the *subscapularis* (IR fatigue), through the evolution of the Kinematic parameters. Machine learning algorithms, such as support vector machines, have already shown interesting results in discriminating the kinematic parameters in attention deficit hyperactivity disorder and control group children (Faci et al., 2020c). The use of these algorithms could be of interest for a differentiation of the type of neuromuscular fatigue (ER or IR fatigue).

### Intra-Participant Follow-Up

Our study showed that an individual monitoring of fatigue is possible using a tablet. It is clinically relevant as pre- and post-fatigue variations are different regarding the type of fatigue, the participant and the test performed. This can be explained by the task dependency of fatigue (Enoka and Stuart, 1992) and the uniqueness of each participant (Chaffin et al., 2006b). In fact, during submaximal muscle contraction, fatigue development depends on the type of fibers activated and on the duration of the contraction (Enoka and Stuart, 1992; Chaffin et al., 2006a). Large variability in duration may come from the inter-subject difference, but also from their ability to generate a maximal force (Edwards, 1981). Different strategies were taken by the participants to counteract the effects of fatigue, which is reflected in the kinematic parameters. This can be observed as well in Figure 5, where behavior differences are illustrated for four velocity profiles. Some participants have a slower general response (Figure 5A), faster (Figure 5C), or not evolving due to fatigue (Figures 5B,D). The participant in Figure 5B was a former high-level swimming athlete, and therefore could be accustomed to shoulder fatigue. The participant in Figure 5D presents many statistical differences in parameters shown by the *U*-Mann–Whitney test. As μ − the longtime delay − significantly decreased and σ − the log response time − significantly increased for this participant, the resulting velocity profile showed little visual differences after fatigue as compared to before. The counterbalanced parameter changes masked the fatigue effect on the velocity profile, pointing out the interest of analyzing the lognormal parameters. Moreover, some participants have a higher μ (Figures 5A,C), meaning a diminution of the neuromuscular system to respond rapidly to a command, whereas some others have a lower value (Figure 5D). To compensate for a lower μ, participants can use a higher σ or *t*_0_. This, respectively, means that the participant will take more time to make the entire movement and that the brain will send the response command later. However, sometimes *t*_0_ significantly decreased after fatigue. The participant reacted faster to the stimulus, as physical exercise can improve someone’s cognitive function (Hillman et al., 2008). Different action plans for drawing strokes were also made: some participants made for example shorter strokes (smaller *D*, Figure 5A) because they had difficulties in executing them, whereas some others made larger strokes (higher *D*, Figure 5C) because they had, for instance, difficulties in stopping them. This is probably due to motor variability as movement is reorganized to prevent the apparition of disorders. In that sense, spatiotemporal muscular recruitment is variable after fatigue, which is assessed here (Falla and Farina, 2007; Srinivasan and Mathiassen, 2012; Yang et al., 2018). This method enables to study the evolution of each parameters and compensations made by participants for a case-by-case study, which is essential for example in personalized top-level athletes training.

### Group Effect of Fatigue

A group effect was noticed from the analyses, signifying that a general pattern is highlighted after a neuromuscular fatigue. This analysis is a first step in the process of using the method in clinics. More studies would be needed to ensure that the parameters evolution highlighted in this study are specific to shoulder fatigue. In the simple strokes, the peripheral system was more impacted after an ER than an IR fatigue. As the time to fatigue was longer in ER, additional mechanisms of fatigue may have been present, such as at the level of the excitation-contraction coupling (Baker et al., 1993), which is then observed in the Kinematic parameters related to the peripheral system. On the contrary, the IR fatigue was perceived harder and may have impacted more the parameters related to the central system. In fact, the action plan of the agonist components of simple strokes is changed, with for example an increase of the time to send the motor command. Moreover, it was noticed that the motor program execution was the most impaired system for most of the tests (Tables 2–4). In fact, the *mode*, *median*, *time delay* were significantly higher after fatigue, meaning a decline in the command velocity (Laniel et al., 2019). However, parameters describing the global state of the neuromotor system, such as the *Nblog* and *SNR/Nblog* have a general trend to increase and decrease, respectively, for the simple strokes and horizontal oscillations after an external rotation fatigue. The evolution of these two parameters reflects a worsening of the motor control quality. In fact, as in Cortes et al. (2014), fatigue impacts the smoothness and motor control of a person. On the other hand, the *SNR* does not seem to change in many cases, only in the simple strokes and triangles after an ER fatigue. As explained in Laniel et al. (2019), the reconstruction of the velocity profiles stops when a 25 dB *SNR* is reached, and adds lognormals until that condition is met. Studying the *SNR* of simple strokes after a Delta-Lognormal extraction might be more appropriate (Plamondon,1995a, 1998; Woch et al., 2011), as it is expected to reconstruct the kinematics with only two lognormals, the *agonist* and *antagonist*. The evolution of the parameters, and especially the ones reflecting the motor program execution, is similar between participants, which is interesting for using the tablet as a clinical tool for fatigue detection.

### Performance of the Tests

As a general overview, simple strokes reveal information about *agonist*/*antagonist* systems. According to Turpin et al. (2011), muscular activity changes after fatigue but the coordination between muscles does not. The same muscles will create the *agonist*/*antagonist* synergy. For this purpose, analyzing and discriminating the two categories of muscles is appropriate. In case of complex tasks (i.e., triangles or oscillations), the distinction between those two systems is meaningless since there is no stop at the intermediate points, only at the end. The use of the speed/accuracy tradeoff tests could provide more information, as it can express further relationships between *agonist* and *antagonist* components and their evolution with fatigue (O’Reilly and Plamondon, 2013). Moreover, central and peripheral system parameters do not show a correlation in simple strokes (*t*_0_ and μ, Table 5), whereas this correlation exits when movements get longer. Participants anticipate them by targeting virtual points (Plamondon et al., 2003; Plamondon and Djioua, 2006). With the independency of parameters in the simple strokes, this test can be specifically used to differentiate a central from a peripheral fatigue.

On the other hand, triangles seem to be more difficult to perform than simple strokes and oscillations. The inter-participant variability may be higher and therefore significant differences can be harder to notice in group studies. However, as depicted in Table 1, individual changes are detectable on triangles, and can therefore be used for extensive studies. A more specific method for extracting triangles may be more adapted, as they have the lowest number of lognormals retained compared to other tests (Figure 3). As a matter of fact, it also depicts the importance of performing more repetitions.

In addition, studying larger movements, such as the oscillations seems more efficient to detect fatigue, as they depict a more biomechanical movement. Nevertheless, a compensatory effect between participants is noticed for the vertical oscillations. As participants adopted different postures to execute the movements, the individual kinematic might have been affected (Fuller et al., 2009). It would have been interesting to record the overall kinematic of the upper-body. In fact, a test performance is often the same pre- and post-fatigue, but strategies to perform the tests are different (Côté et al., 2002; Emery and Côté, 2012).

### Opening

In a wider context, the Sigma–Lognormal model seems appropriate to study fatigue at different levels of the body, whether it is the upper limb or lower limb. In fact, fatigue results in deficiency in motor control and motion changes due to a modification at different biological levels of the human body physiology (Enoka and Stuart, 1992; Gandevia, 2001; Cortes et al., 2014). That is why, it is expected that any other impairment in the body, due to fatigue, could be detectable by a similar method. Also in Cowley and Gates (2017), it has been noticed that finger or shoulder fatigue affect movement coordination in different manners. In this way, we think that it would be possible to discriminate fatigue from different parts of the body and parameters from the theory could reflect those changes. By performing wider movements with the use of a white board (Fischer et al., 2014), or by registering them in 3D (Schindler et al., 2018), it would then probably be easier to discriminate them. The use of a board seems interesting, as the system would remain easy to use. As the Kinematic Theory describes fine motor control and is suitable for many end effectors [such as fingertips, head (Lebel et al., 2018b), eye movements (Plamondon,1995a) etc.], the use of markers directly on the studied articulation would be interesting to complete analyses.

## Conclusion

This study highlights that shoulder neuromuscular fatigue is detectable in healthy active adults with the use of a digitizing tablet and the Kinematic Theory. The type of fatigue (central or peripheral) and the location of central fatigue (preparation or conduction time) are distinguishable through the parameters extracted from handwriting. An individual monitoring is relevant to determine the compensatory reactions made by each participant to counteract the effects of fatigue. Overall, common patterns in the parameters evolution are noticeable and are significant for clinical studies. Parameters having a more global approach, such as the *mode*, *median*, *time delay* tend to increase after fatigue, whereas the *SNR/Nblog* tends to decrease. We also observed that all handwriting tests were sensitive to fatigue. Nevertheless, the simple strokes test could discriminate between the central and peripheral systems independently and between the *agonist/antagonist* systems, and the oscillations test is the most effective to detect shoulder fatigue.

## Data Availability Statement

The raw data supporting the conclusions of this article will be made available by the authors, to any qualified researcher interested in collaborating with our team.

## Ethics Statement

The studies involving human participants were reviewed and approved by Research Ethics Committee of Polytechnique Montréal (CER-1819-23 v.3). The patients/participants provided their written informed consent to participate in this study.

## Author Contributions

AL, RP, and MB contributed conception and design of the study. AL recruited the participants and performed data collection, data formatting, and statistical analyses. AL, RP, and MB interpreted the results. AL wrote the first draft of the manuscript. RP and MB critically reviewed it. The final version was approved by all authors.

## Conflict of Interest

The authors declare that the research was conducted in the absence of any commercial or financial relationships that could be construed as a potential conflict of interest.
