# Supplementary material for: Central and Peripheral Shoulder Fatigue Pre-screening Using the Sigma–Lognormal Model: A Proof of Concept
Source: Front Hum Neurosci. 2020 May 19;14:171. doi: 10.3389/fnhum.2020.00171 (PMC7248386; doi:10.3389/fnhum.2020.00171)
Supplement: Supplementary file 1 [file Table_1.DOCX]

Supplementary Material

Table 1 | Resume of the parameters extracted and their signification

| **Input level: central system** | |
| --- | --- |
| **t_0_** | It is the time that takes the brain to perceive the stimulus and emit the command to the musculoskeletal system. It refers to the moment when a population of neurons sends a motor command, it occurs after the audible stimulus is perceived and the motor command prepared. |
| **Δ(t_0_)** | It reflects the rhythmicity of an input command. It represents the time elapsed between two successive *t_0_* and is used in the oscillations only. |
| **D** | It corresponds to the distance covered by the resulting lognormal. |
| **θ_s_** | It is the starting angle of the lognormal. |
| **θ_e_** | It is the ending angle of the lognormal. |
| **Timing properties of the neuromuscular system: peripheral system** | |
| **μ** | Also known as the logtime delay, it represents the time taken to reach half of the distance movement on a logarithmic scale. It corresponds to the rapidity of a reaction to a command by a system. |
| **σ** | Also known as the logresponse time, it represents the time taken from the neuromuscular system to respond to a command on a logarithmic scale. It is also linked to the movement duration and is a measure of the asymmetry of the lognormal. |
| **Global state of the neuromotor system** | |
| **Nblog** | It is the number of lognormals required to reconstruct the velocity profile of the movement. |
| **SNR** | It is the measure of the quality of the movement reconstruction. |
| **SNR/Nblog** | It is a performance criterion and represents the motor control fluency of a gesture. The lognormality principle predicts that the ideal movement converges toward a lognormal profile. When the SNR/Nblog is higher, the movement is more similar to the ideal one, as postulated by the lognormal behavior. |

| **Motor program execution** (Δ(t_0_) is used instead of t_0_ for the calculus in the oscillations) | |
| --- | --- |
| **Mode** | It is the time at which the maximum value of the lognormal impulse response is reached.  $M=t_{0}+ e^{\mu-\sigma^{2}}$ |
| **Median** | It corresponds to the time at which the half value of the integral under the lognormal curve (50% of the covered distance) is reached.  $m=t_{0}+ e^{\mu}$ |
| **Time delay** | It represents the rapidity of a neuromuscular system to respond to a command.  $\bar{t}=t_{0}+ e^{\mu+0.5\sigma^{2}}$ |
| **Response time** | It is a measure of the spread of the impulse response.  $s=\left( \bar{t}-t_{0} \right)\sqrt{\left( e^{\sigma^{2}}-1 \right)}$ |
| **Asymmetry** | It characterizes the shape of the lognormal.  $A_{c}=1-e^{-\sigma^{2}}$ |
| **Other parameters** | |
| **Reaction time** | It is the time needed to start the movement after a stimulus. In the present study, it was computed as the time required to reach 10% of the maximal velocity during the test |
| **Command propagation** | It is the duration of the command propagation  $CP=RT-t_{0}$ |
